# Is It Feasible to Use CMV-Specific T-Cell Adoptive Transfer as Treatment Against Infection in SOT Recipients?

**DOI:** 10.3389/fimmu.2021.657144

**Published:** 2021-04-23

**Authors:** Estéfani García-Ríos, Marcos Nuévalos, Francisco J. Mancebo, Pilar Pérez-Romero

**Affiliations:** National Center for Microbiology, Instituto de Salud Carlos III, Majadahonda, Spain

**Keywords:** cytomegalovirus, CMV-specific immune response, T-cell adoptive transfer, CMV treatment, cellular therapy

## Abstract

During the last decade, many studies have demonstrated the role of CMV specific T-cell immune response on controlling CMV replication and dissemination. In fact, it is well established that transplanted patients lacking CMV-specific T-cell immunity have an increased occurrence of CMV replication episodes and CMV-related complications. In this context, the use of adoptive transfer of CMV-specific T-cells has been widely investigated and applied to Hematopoietic Stem Cell Transplant patients and may be useful as a therapeutic alternative, to reconstitute the CMV specific T-cell response and to control CMV viremia in patients receiving a transplantation. However, only few authors have explored the use of T-cell adoptive transfer in SOT recipients. We propose a novel review in which we provide an overview of the impact of using CMV-specific T-cell adoptive transfer on the control of CMV infection in SOT recipients, the different approaches to stimulate, isolate and expand CMV-specific T-cells developed over the years and a discussion of the possible use of CMV adoptive cellular therapy in this SOT population. Given the timeliness and importance of this topic, we believe that such an analysis will provide important insights into CMV infection and its treatment/prevention.

## Introduction

Viral infection, including cytomegalovirus (CMV), BK virus and Epstein-Barr virus, remains a major cause of morbidity and mortality in immunocompromised individuals (1–5). While in immunocompetent individuals latent CMV infection is controlled by the immune system (6), in transplant recipients, both hematopoietic stem cell (HSCT) and solid organ transplantation (SOT), CMV infection is one of the main infectious complications. CMV seropositive allogeneic HSCT patients presents the highest risk of recurrent infections, followed by CMV seronegative SOT recipients that receive a graft from a seropositive donor (R-/D+), HIV patients, and patients who have received T-cell depletion therapies (alemtuzumab, antithymocyte globulin, or post-transplant cyclophosphamide) (6, 7). The incidence of CMV reactivation/reinfection in SOT is 16–56% (8–12), with a median value of 30%, while in HSCT has been reported to be 30–70%, with a median value of 37% (13–15). In addition to the direct effects of CMV proliferation causing viral syndrome with clinical manifestations such as gastroenteritis, pneumonitis, hepatitis, uveitis, retinitis, encephalitis and graft rejection, CMV infection also cause indirect effects related with increased incidence of graft rejection and opportunistic infections or decreased recipient survival (16–18).

Cell mediated immune response is considered the most important arm of the immune system against CMV infection with increasing evidences demonstrating a role of CMV-specific T-cells in protecting from infection, which can contribute to improve clinical care after transplantation (19–27).

A few authors have suggested the importance of monitoring patient’s CMV-specific immunity using standardized tools for individualizing the risk of CMV infection after transplantation (21, 28–31). Thus, using both immunological and virological patient monitoring may provide a wider knowledge of patients’ clinical situation that may facilitate clinical decisions during follow-up of SOT recipients (32).

Although the antiviral drugs to treat CMV infection have highly improved during the years, there are still some issues associated with the use of the available antivirals (ganciclovir, foscarnet, cidofovir and more recently letermovir) such as undesirable side effects (nephrotoxicity) and selection of resistance mutations in addition to the high cost. Consequently, strong efforts have been made to search for new therapeutic approaches (33).

In this context, the use of cellular therapy may be useful to reconstitute the CMV specific T-cell response and to control CMV viremia in SOT recipients. Here we provide a synthesis of recent data regarding the impact of using CMV-specific T-cell adoptive transfer on the control of CMV infection in SOT recipients, the different approaches to stimulate, isolate and expand CMV-specific T-cells developed over the years and a discussion of the possible use of CMV adoptive cellular therapy in these patients.

## Use of Adoptive Transfer of CMV-Specific T-Cells in the Context of Solid Organ Transplantation

The use of CMV-specific T-cell adoptive transfer is currently being evaluated for clinical application, with promising results as a treatment for CMV infection and disease in ulcerative enteritis in primary immunodeficiency (34) or in pediatric retinitis caused by CMV (35).

In the context of transplantation, CMV-specific T-cell transfer has been widely investigated and applied to Hematopoietic Stem Cell Transplant (HSCT) patients, both prophylactically, to reconstitute protective antiviral immunity, and as a treatment in patients with refractory CMV infection (36–38). In contrast, in SOT recipients it has been less investigated probably due to the T-cell response attenuation produced by the administration of the immunosuppressive therapy. In addition, SOT recipients may not tolerate donor-derived cytolytic T lymphocytes (CTLs) due to the activation of cytokine-mediated stimulation of the alloreactive T-cells causing direct alloimmune injury (39, 40).

Few authors have explored the use of T-cell adoptive transfer in SOT recipients during the last decade (**Table 1**). In 2009, Brestrich et al. (41) performed a study in a lung transplanted recipient with a severe and persistent CMV pneumonia resistant to ganciclovir and foscarnet. Patient´s peripheral blood mononuclear cells (PBMCs) were stimulated with overlapping peptide pools covering the whole protein IE-1 and pp65 and CMV-specific INF-γ positive cells were subsequently selected and infused. The patient was treated with two infusions of 1 × 10^7^/m^2^ CMV‐specific T-cells. After the first infusion, the patient developed an overall improvement, with a decrease of the viral load and pneumonia symptoms and an increase of the CMV-specific T-cell levels. Four weeks after the first infusion, a second infusion was administered due to a worsening of the disease, testing positive for CMV. However, the patient died due to graft failure with a negative biopsy for CMV antigen (41).

**Table 1:** List of available works of CMV-specific T-cell transfer in SOT.

Method	Organ and D/R status	Number of infused cells	Number of infusions	Cell line phenotype	Stimulation method	Post-infusion clinical outcomes
Direct selection by IFN-γ capture Brestrich et al. (41)	1 Lung +/+	Fresh 1 × 10^7^ T-cells/m^2^	2	95% CD3+ cells with 2.7% and 92.3% CD4+ and CD8+ cells. No CD16+ natural killer cells and only 0.1% CD19+ B cells	Overlapping IE-1/pp65 peptide pools	No side effects occurred after the infusion. The number of CMV-specific T-cells increased, while viral load decreased. The patient died from graft failure
*Ex vivo* expansion from a third party donor (43)	1 Kidney +/−	Frozen 1.6 x10^7^ T-cells/m^2^	1	16.6% CD4+ and 79.4% CD8+ cells	Overlapping pp65 peptide pool	The patient developed a mild fever but no other adverse effects were noted and within 4 months his CMV viral load decreased from >5×10^6^ copies to 682 copies/mL and remained controlled up to 1 year
Autologous *Ex vivo* expansion (46)	1 Lung +/−	Fresh 3 × 10^7^ T-cells	4	82.6% CD3+ cells, including 14% CD4+ and 73.8% CD8+ cells	PBMC coated with HLA class I-restricted CMV epitopes	Decrease in viral load. No graft rejection
Autologous *Ex vivo* expansion (47)	1 Lung +/−	Frozen two of 1.9 x10^7^ cells and one of 22.2 x 10^6^ T-cells	3	Two first infusions 41.6% CD8+ cells Third infusion 4.43% CD8+ cells	HLA Class I restricted epitopes from pp65, pp50 and IE-1	The patient did not have any documented rejection or acute change in lung function after the T-cell infusions but finally died due to clinical complications unrelated to CMV
Autologous *Ex vivo* expansion (48)	13 kidney, 8 lung and 1 heart +/− +/+ −/−	Frozen 22.2-245 × 10^6^ T-cells	6	20% CD4+ and 70% CD8+ cells	HLA class I– and class II–restricted epitopes from pp65, pp50, IE-1, gH, and gB	None of the patients who received adoptive CMV-specific T-cell therapy showed treatment-related grade 3, 4, or 5 adverse events. Reduction or resolution of CMV reactivation and/or disease and improved response to antiviral drug therapy

Since then, a number of authors have explored the potential of T-cell adoptive transfer as a therapy in SOT recipients (42). A renal transplant recipient (D+/R-) with refractory CMV infection received partially HLA-compatible (at three of six HLA loci A, B and DRB1) CMV-specific T-cells at a dose of 1.6 × 10^7^ T-cells/m^2^, successfully generated from a third donor. Nineteen days following the infusion, a fifty fold decreased of the CMV DNA viral load was observed and plasma exchange was ceased due to resolution of hematological features of thrombotic microangiopathy (platelets 269 × 109/L, LDH 369 IU/L, no red cell fragments on blood film). Patients was discharged from hospital four weeks after the infusion (43). The authors highlighted the effective application of CMV-specific CTLs from third donors, suggesting that creating donor cell-banks could be useful as a therapeutic alternative in SOT recipients (43–45). In a later study, the same group successfully expanded autologous CMV-specific T-cells from a seronegative recipient that received a seropositive lung allograft and that developed a CMV disease due to ganciclovir resistant CMV infection (46). CMV-specific T-cells were isolated and stimulated with autologous PBMCs coated with HLA class I-restricted CMV peptide epitopes, based on patient´s HLA class I typing. The *in vitro* expanded T-cells showed an increase in HLA epitopes (A1, B7 and B35) and in the proportion of IFN-γ+ CD107a+ cells that indicates the granule-dependent (perforin/granzyme) pathway of cytotoxic CD8+ T-cells. The patient received four infusions of 3 × 10^7^ autologous T-cells. After the infusion of the *in vitro* expanded T-cells no adverse events occurred, the CMV viral load became undetectable, the patient’s usual immunosuppression regime was resumed, hepatic and bone marrow function remained normal with no evidence of acute rejection. These results indicated that adoptive therapy can contribute to immune control of CMV infection (46). Pierucci et al. (47) employed autologous T-cell transfer in a seronegative lung transplant recipient with a ganciclovir and foscarnet resistant CMV infection, who also developed cidofovir-related nephrotoxicity. Cells were obtained from patient´s peripheral blood and expanded using epitopes of synthetic HLA-compatible peptides (pp65, pp50 and IE-1). Around 42% of the obtained CD8+ T-cells were CMV-specific and T-cells were restricted to three HLA Class I alleles: HLA-A1, HLA-B7 and HLA-B8. The patient received 2 infusions (1.9 x 10^7^ T cells/infusion) 2 weeks apart, with no side effects and with low CMV titers during two months after which a relapse of the viral load occurred. The patient received a third infusion (22.2 x 10^6^ T-cells) showing some therapeutic benefit, with further significant reduction in CMV titers, which was maintained for 2 months. The patient did not have any documented rejection or acute change in lung function after the T-cell infusions. However, the patient died due to clinical complications unrelated to CMV infection (47).

The most ambitious study carried out to date was performed in a cohort of 21 SOT recipients (13 kidney, 8 lung and 1 heart) who developed recurrent ganciclovir resistant CMV infections. Thirteen of these patients (8 D+/R-, 3 D+/R+ and 2 D-/R-) were subjected to T-cell (ranging from 22.2-245 × 10^6^ T-cells) adoptive transfer receiving a maximum of 6 doses one of which discontinued therapy after a single dose. Adverse events attributable to T-cell infusion were grade 1 or 2 (fatigue and malaise) with no adverse events associated with a change in the graft status. Eleven of the 13 showed objective improvement in their symptoms including a reduction (with a median drop of 1.2 × 10^3^ CMV copies/mL) or resolution of CMV reactivation and resolution of CMV disease symptoms. In addition, the use of antiviral drug therapy was either completely stopped (in 5 of 11 patients) or significantly reduced (in 6 of 11patients). Evidences of immunological reconstitution was associated with control of viremia (48).

Based on these promising results, several clinical studies are currently been conducted: (i) A clinical trial (NCT03665675) including 20 patients, both HSCT recipients and SOT recipients is been conducted, to study the effect of transferring allogeneic CMV-specific T lymphocytes on CMV infection or reactivation. The first results will be available at the end of 2021. (ii) A clinical trial (NCT02779439) with 25 patients enrolled, to elucidate the biological efficacy of therapeutically administered most closely HLA-matched third-party donor-derived specific cytotoxic T lymphocytes (CTLs) targeting CMV, following allogeneic blood or marrow stem cell or SOT. (iii) A clinical trial (NCT04364178) including 25 patients assessing whether partially matched, ≥2/6 HLA-matched, viral specific T-cells have efficacy against CMV in subjects who have previously received any type of allogeneic HSCT or SOT. (iv) A clinical trial (NCT03266640) with 20 participants investigating the therapeutic role of CMV CTLs in children, adolescents and young adults (CAYA) with refractory CMV infection post allogeneic HSCT or SOT.

Together these results suggest that, although there is still space for improvement, the use of CMV-specific T-cell adoptive transfer is promising in SOT recipients with limited options for CMV-infection treatment.

## Cellular Therapies Available

**Figure 1:**
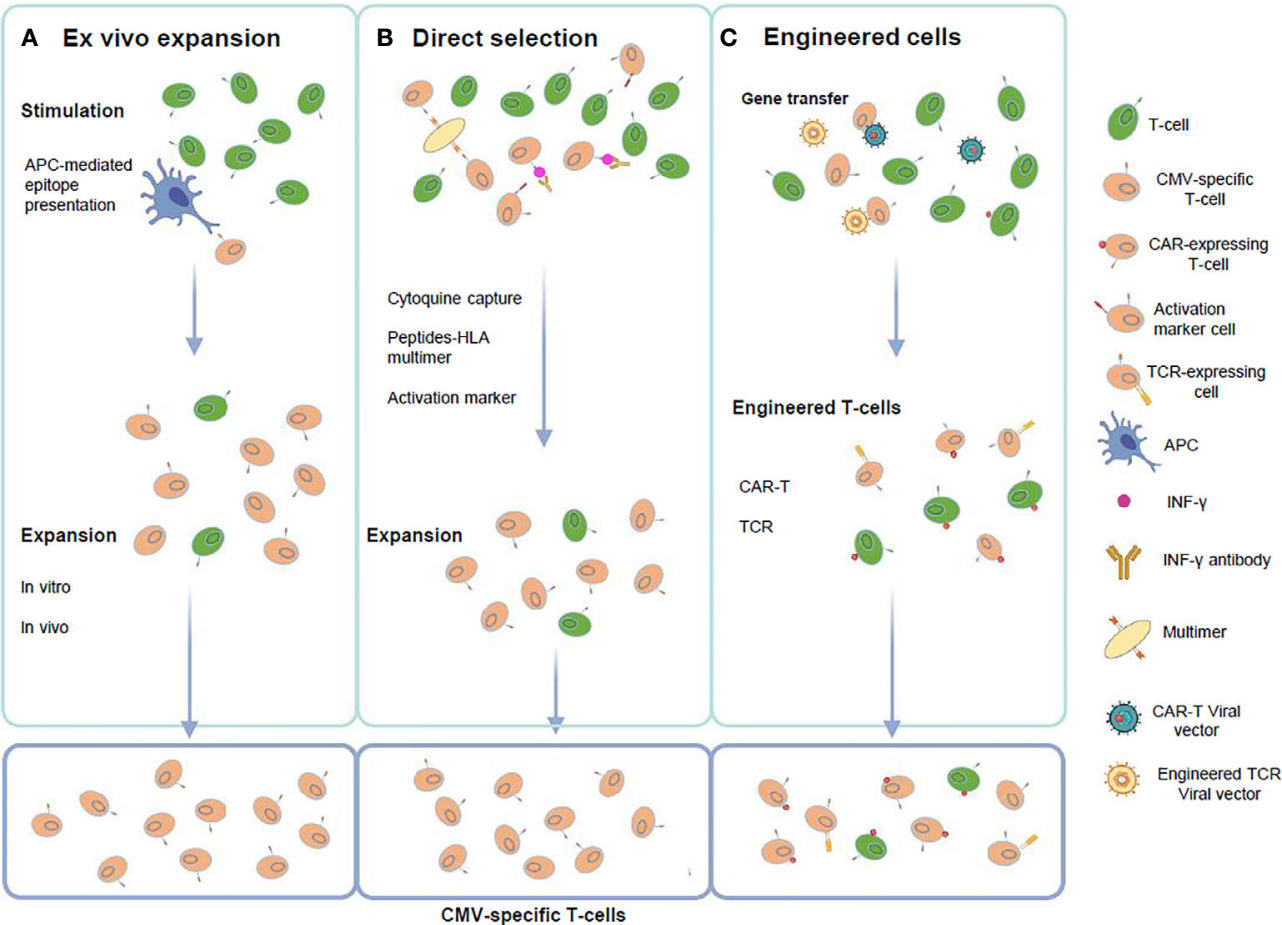
Strategies for the generation of CMV-specific T-cells. **(A)** *Ex vivo* T-cell expansion requires the *in vitro* stimulation and expansion of T-cells using APCs presenting viral peptides or proteins. **(B)** Direct selection employs virus-derived peptide specific multimers in the setting of a HLA class-I molecule, viral antigen T-cell stimulation followed by cytokine expressing T-cell selection using antibody coated immunomagnetic beads or activation marker selection based on the detection of specific surface molecules that are selectively expressed or strongly up-regulated after T-cell activation. **(C)** Genetic manipulation requires gene transfer of high affinity CMV-specific T-cell receptors (TCR) or chimeric-antigen receptors (CAR) to change specificity of T-cells to CMV antigens. This figure was created using BioRender.com.

During the last years a better understanding of the CMV-specific T-cell immunology such as the conserved T-cell epitopes (49), has led to the improvement of the methods for *ex vivo* T-cell culture (50). In addition, rapid tests to evaluate the effector function of the CMV-specific T-cells have become available (51, 52). In this section, we describe the features of the methodologies available to generate CMV specific T-cells, which are summarized in **Figure 1** and **Table 2**.

**Table 2:** Characteristics of the T-cell therapies available.

Method	System	Advantages	Disadvantages
***Ex vivo* expansion**		No restricted by HLA type; small blood volume required; naïve donor can be used; generation of polyclonal T-cells	Extensive culture period; seropositive donors required
**Direct Selection**	pMHC multimer	No needed extensive *ex vivo* manipulation and undergo rapid expansion *in vivo*	Restricted by HLA type and streptamer; seropositive donors required; high frequency of specific T-cells needed; select for a limited repertoire of CD8+ cells
	Cytoquine capture	No needed extensive ex vivo manipulation and undergo rapid expansion *in vivo*; not restricted by HLA type; isolation of polyclonal CD4+ and CD8+ cells	Requires seropositive donors; large blood volumes needed
	Activation marker	Rapid detection and enrichment of T-cells; broader repertoire of antigen-specific T-cells; Compatible with other assay formats; not restricted by HLA; not needed previous information of immunodominant epitopes; no specialized APC such as dendritic cells are needed	Time-consuming and difficulty to isolate and expand functional cells; identification of novel T-cell epitopes often requires screening of a high number of epitopes
**Genetically engineered cells**	CAR-T	Recognize antigens in an HLA-independent manner; target conserved and essential epitopes; infused to a broad range of patients irrespective of HLA	Only surface antigens can be targeted; restricted by epitope; expensive; Several toxicities
	TCR-T	Wider range of targets; high affinity for specific antigens through genetic engineering; strong activation when a small amount of antigen is present; use of natural T-cell signaling mechanisms	Expensive; time- and labor-consuming; MHC restricted and depends on presentation by MHC molecules to recognize targets and activate T cell function; risk of hybridization (mismatch) between exogenous and endogenous chains
			

### T-Cell Expansion

To successfully generate and expand CMV-specific T-cells, it is crucial to define the most immunogenic epitopes used by the antigen presenting cells (APC) to promote the activation and proliferation of peptide-specific T-cells (53). A large number of antigens expressed at different stages during viral replication participate in the activation of both CMV-specific CD8+ and CD4+ T-cells, known to mediate the immune response against the virus (50). IE-1 and pp65 proteins are two of the most immunodominant CMV antigens and have been widely used to stimulate the CMV-specific immune response (50, 54–56).

Different approaches have been carried out for *in vivo* expansion and generation of CMV-specific T-cells (57). In the initial studies, CMV-specific CD8+ T-cell clones were generated by stimulating donor peripheral blood mononuclear cells (PBMC) with CMV-infected fibroblasts (23). However, this approach was discontinued because of the risk of producing infection in patients. Later, CMV lysates or pp65-NLV peptide were used to stimulate CMV-specific T-cells (58–60). Using the pp65-NLV peptide only stimulated adoptive immunity against a single viral epitope (50) and its application may be limited for HLA-A2 patients/donors (60). To overcome this problem, “poly-specific” products targeting multiple antigens were generated by incubating allogeneic T-cells *in vitro* with clusters of 15-mer peptides spanning the entire pp65 antigen to generate CMV-specific oligoclonal T-cells (61). Adoptive transfer of the oligoclonal T-cells were able to eliminate viremia, and infused cells persisted for up to two years (61, 62).

The improvement of the methodology for *ex vivo* expansion has reduced the presence of alloreactive or naive T-cells in the final product (63). In addition, T-cell *ex vivo* stimulation and expansion requires a small blood volume to establish the T-cell culture, making possible the generation of CMV-specific T-cells from low levels of circulating T-cells and naive donor sources (51).

### Direct Selection Using Specific Peptide–MHC (pMHC)

Using pMHC multimers allows to isolate T-cells based on the T-cells receptor (TCR) ability to bind a complex mixture of peptide-loaded recombinant HLA molecules (53). Since this method is restricted by HLA type, a previous knowledge about the immunodominance of the epitopes is necessary. HLA-peptide tetramers from pp65 and IE-1 proteins have been previously used to select CD8+ T-cells that were further isolated using magnetic beads (64).

This method allows to reduce the time and improve the quality of the final product, minimizing alloreactivity (57). However, the main disadvantages of this technique are related with the limitation of the method to isolate only CD8+ or CD4 T-cell populations, and the irreversibility of the binding that can cause changes in the T-cell phenotype, leading to functional alterations of the purified T-cell population (such as TCR internalization, activation, overstimulation and cell death) (65–67). It has been shown that pMHC multimer binding interferes with the functional status of epitope-specific T-cell population *in vivo*, causing epitope-specific tolerance in a dose-dependent manner (68, 69). This intrinsic characteristic of pMHC multimer binding substantially limits the clinical application of this technology.

This issue has been further solved with the development of the Streptamer technology in which the binding of the HLA peptide and the antigen-specific TCR is reversed, by competing with a molecule that causes the Streptamer to monomerize, causing no alteration of the phenotype or the functional status of the T-cells (70–72). However, the selected T-cells are limited by the HLA restriction imposed by the Streptamer, and this may be a limitation for CD8+ T-cells survival when CD4+ T-cells are absent (73). Some authors have used this new technology to isolate CD8+ T-cells from CMV seropositive donors, demonstrating both immune reconstitution, as well as antiviral safety and efficacy after HSCT (74, 75). The results obtained with this technology are promising however, further studies are necessary to demonstrate efficacy in SOT recipients.

In the context of SOT, p-MHC multimers has been previously used using autologous T-cells harvested from lifelong immunosuppressed patients (while healthy donors were used in HSCT). In these patients, deficiencies in T-cell differentiation, longevity, as well as the use of immunosuppressive regimen can affect to long-term survival of the transplanted cells limiting its use for adoptive therapy (76). The associated challenges of this method could be minimized by using partially HLA-matched CMV-specific T-cells obtained from a third party donor (43). This approach was shown to be safe to treat CMV infection in SOT patients, however, more research is needed (43).

### Direct Selection Using Cytokine Capture System (CCS)

CMV-specific T-cells can also be selected using IFN-γ cytokine capture system (CCS), a rapid assay that allows to select and enrich CD8+ and CD4+ INF-*γ* secreting T-cells that have been previously stimulated using viral antigens (77). This strategy allows T-cell selection that in contrast with pMHC has no HLA restriction and as an additional benefit, stimulating and capturing a polyclonal population of CD4+ and/or CD8+ T-cells depending on the antigen used for stimulation, not achieved using the Streptamer strategy. Different authors have successfully isolated functional CMV-specific T-cells using this method. Two of these studies stimulated donors PBMC using pp65 that were administrated to patients after HSCT who were able to expand the CMV-specific T-cells and reduced the CMV load in blood (78, 79). More recently, Kim et al. (80) used the automated CliniMACS Prodigy platform to generate pp65-specific CTL that exhibited functional activity, including efficient proliferation, sustained antigen-specific IFN-γ secretion, and cytotoxicity against pp65-pulsed target T-cells. Although little clinical experience is available, this approach has the potential to be applicable to any type of patients with a clinical emergency due to CMV-related diseases including SOT recipients (80, 81).

Other selection strategy is to isolate and enrich activated viral-specific T-cells after antigen stimulation based on the detection of specific surface molecules that are selectively expressed or strongly up-regulated after T-cell activation, such as CD25, CD69, CD137 and CD154 (82–84). In this sense, several publications have shown results using CD137 as a specific activation marker due to its predominant expression on T lymphocytes after activation, including CD8+ and CD4+ cells (85, 86). This approach allows simultaneous targeting of antiviral T-helper and effector cells. Other data showed the feasibility of isolating CMV-specific T-cells from PBMCs through the use of CD25 and CD154 activation marker expression (82, 87). However, as both markers are predominantly expressed in CD4+ T-cells, these strategies do not allow the enrichment of CD8+ cytotoxic T-cells.

### T-Cell Generation Using Activation Marker and Engineered T-Cells (CAR, TCR)

Other interesting strategies based on the successful performance for cancer treatment (88–90) is the gene modification of patient’s lymphocytes with tumor-specific T-cell receptors (TCRs) or chimeric antigen receptors (CAR). The generation of autologous CAR T-cells which has also been explored as immunotherapy against CMV (91, 92, *93*) enables antigen recognition in a MHC independent manner and can be designed to specifically target conserved and essential epitopes of the selected antigen (94), overcoming pathogen escape mechanisms. In a nutshell, CAR consists of a defined antigen-binding domain represented by a single-chain fragment variable (scFv) antibody, an extracellular spacer region, a transmembrane domain, and an intracellular domain that triggers T-cell activation, mainly by the T-cell receptor signaling domain CD3ζ (94). Several groups have recently generated gB-targeted CAR T-cells using scFvs derived from gB-specific NAb antibody (SM5-1) fused to CARs with 4-1BB (BBL) or CD28 (28S) costimulatory domains and subcloned into retroviral vectors (95, 96). In a recent study, CD4+ and CD8+ T-cells obtained from blood or cord blood of CMV-seronegative donors were transduced showing efficacy in preclinical models (96). Further clinical studies will be necessary to demonstrate *in vivo* efficacy.

The other TCR strategy uses heterodimers integrated by alpha and beta peptide chains to recognize specific polypeptide fragments presented by MHC complexes. While CAR-T-cell therapy identifies exclusively antigens located in the cell surface, TCR can also recognize intracellular antigenic fragments presented by MHC molecules (97). However, TCR T-cell therapy is restricted to MHC presentation, which represents a limitation of the strategy. The main goal of TCR T-cells is to modify the TCR binding to the pathogen antigens. Naturally, the affinity of TCRs for the pathogen antigens is very low, which difficult the recognition. To overcome this problem, modifications of the TCR using genetic engineering technology has been able to enhance the specificity and affinity of the recognition of the antigens by T-cells (98).

### Cell Therapy Limitations and Alternatives in SOT Patients

The intensity and long-term immunosuppression requirement to prevent allograft rejection pre-disposes SOT recipients to a wide range of viral complications (1). In addition, antiviral treatment can generate side effects such as nephrotoxicity (99), and the selection of drug-resistant mutant CMV strains (100), limiting treatment capability in SOT recipients. Based on these limitations, cell therapy may be an appropriate and effective alternative antiviral treatment. However, as pointed out previously, deficiencies in T-cell differentiation and lifelong immunosuppression can affect to long-term survival of the transfused cells, interfering in the antiviral functionality and limiting its use for adoptive therapy in SOT recipients (76). Here, we analyze the alternatives available to overcome these limitations.

Different authors have demonstrated that *in vitro* generated CMV -specific CTL are highly sensitive to immunosuppressive drugs (such as cyclosporin A and FK506) impairing the production of effector cytokines (101, 102). A possible solution in order to overcome this problem, is to genetically modify the *in vitro* generated CTL to confer resistance to these drugs (103, 104). Alternatively, decreasing patient´s immunosuppression during a period post-infusion may allow the expansion and functionality of the CMV-specific T-cells. As an example, Macesic et al. used third-party T-cells to infuse a kidney transplant patient who had ganciclovir resistant persistent CMV viremia, and decreased the levels of immunosuppressive drugs. A significant decrease of the patient CMV DNA viral load, from >5x10^6^ copies to 682 copies/mL, was observed within 4 months after transfusion and remained controlled up to 1 year, leading to clearance of the infection (43). These results suggested that the use of third-party CMV-specific T-cells could be used in patients that admit a reduction of the immunosuppression regimen without compromising the allograft stability.

Another limitation is associated with deficiencies of T-cell differentiation in SOT recipients receiving immunosuppression. Most of the studies have used the viral antigens UL123 (IE1) and UL83 (pp65), known to promote a strong T-cellular response, for T-cell ex vivo stimulation to generate CMV-specific oligoclonal T-cells. Few studies have provided information regarding the cell mediated response to other viral multiple antigens in addition to IE1 and pp65 (54, 105–107). Thus, efforts should be made to promote the generation of CD8+ and CD4+ T-cells displaying multiple polyfunctional effector functions that may be more effective in controlling CMV infection (50, 54–56).

As previously mentioned infusion of donor derive T-cells from donors may also transfer alloreactive T cells in numbers sufficient that could trigger episodes of rejection, particularly if the donor and the host differ in one or more HLA alleles, due to sensitization to specific non-self HLA alleles present on the donor T-cells. A way of assessing this issue is to extensive culturing T cells or even establishing T-cell clones to eradicate alloreactive T cells but may also result in replicative senescence of the *ex vivo*-manipulated virus-specific T cells (108).

The creation of third-party cell banks as well as third party donor registries has emerged as a new possibility of treatment that employs T-cells derived from partially HLA-matched third-party donors (109). The use of this method allows to achieve a rapid “off the shelf” product that could be used in a broader range of patients. Furthermore, it offers the potential advantage of targeting multiple viral epitopes rather than a monospecific approach, potentially increasing the antiviral effect (109). Over the past years third party donor T-cell banks have been established. Such banks permit selection of T-cells on the basis of HLA allele phenotype, viral specificity and HLA restriction, which may provide distinct advantages, particularly in the treatment of HLA non-identical recipients. Although it is still under study, the obtained results to date are highly promising.

## Future Directions and Perspectives

CMV is a major cause of severe complications in SOT recipients such as graft loss especially in patients that develop CMV infection with antiviral refractory CMV strains (110, 111). The period early after the transplant is considered critical due to the high risk of infections associated with a high incidence of CMV (42). The role of CMV-specific T-cell immune reconstitution after SOT have demonstrated several benefits, including lower risk of CMV infection and graft rejection. Thus, the development and improvement of new CMV-specific T-cell transfer based therapies could be a useful to adjust the therapeutic interventions (112–114). However, despite the increasing interest on adoptive CMV specific T-cell transfer, most of the information available comes from studies in HSCT recipients (23, 53, 115). Only few reports including a small number of SOT recipients have used T-cell adoptive immunotherapy as a treatment of CMV infection or disease (41, 43, 46–48). These studies enrolled SOT recipients that previously failed to conventional treatment, with low survival rate. Although promising results were obtained, further development have been limited due to difficulties of T-cell expansion in SOT that are receiving immunosuppressive regimens, and the risk of graft rejection after T-cell administration. One possible approach to overcome these limitations is generating ready to use third-party CMV-specific T-cell banks to ensure the availability of well characterized the T-cell products (57, 116). In addition, better results should be obtained using T-cell adoptive immunotherapy in SOT recipients that had optimal clinical outcomes. Results from the ongoing clinical trial analyzing the safety and feasibility of administering CMV specific- CTLs from haploidentical donors in transplant patients would be of importance to implement T-cell adoptive therapy in SOT recipients.

## Conclusions

Recent studies have significantly increased our knowledge about the protective role of CMV-specific T-cell immune response against CMV infection and disease. And thus the use of T-cell adoptive therapy may help to restore the CMV-specific immunity for preventing CMV infection in addition to serve as a treatment for CMV infections in SOT individuals who do not respond to conventional therapies, such as patients infected with antiviral resistant strains with no alternative treatment available. Recent findings regarding the development of new techniques to select, isolate and enrich functional CMV-specific T-cells and the possible generation of third party donor cell banks may help to use CMV-specific adoptive transfer as an alternative therapy for SOT recipients. However, further work is clearly needed in order to fully understand and assess the clinical utility of these techniques in SOT recipients.

## Author Contributions

MN and FM: writing and revision of the manuscript. EG-R: conceptualization, writing, revision, and editing of the manuscript and supervision. PP-R: project funding and administration, conceptualization, writing, revision and editing of the manuscript, and supervision. All authors contributed to the article and approved the submitted version.

## Funding

This study was supported by the Spanish Ministry of Science, Innovation and University, Instituto de Salud Carlos III Grant/Award Numbers: PI17CIII-00014 (MPY110/18); DTS18CIII/00006 (MPY127/19); PI20-009 (MPY303/20). This work was supported by Plan Nacional de I + D+i 2013‐2016 and Instituto de Salud Carlos III, Subdirección General de Redes y Centros de Investigación Cooperativa, Ministry of Science, Innovation and University, Spanish Network for Research in Infectious Diseases (REIPI RD16/0016/0009), co-financed by European Development Regional Fund ‘A way to achieve Europe’. EG-R is supported by the Sara Borrell Program (CD18CIII/00007), Instituto de Salud Carlos III, Ministerio de Ciencia, Innovación y Universidades. FM is supported by the PFIS Program (F18III/00013), Instituto de Salud Carlos III, Ministerio de Ciencia, Innovación y Universidades. MN is supported by the FPU program (FPU19/05927), Ministerio de Ciencia, Innovación y Universidades.

## Conflict of Interest

PP-R is the founder and shareholder of Vaxdyn, S.L., a biotechnology company developing vaccines.

The remaining authors declare that the research was conducted in the absence of any commercial or financial relationships that could be construed as a potential conflict of interest.
